# Antioxidant and Antitumor Activity of a Bioactive Polyphenolic Fraction Isolated from the Brewing Process

**DOI:** 10.1038/srep36042

**Published:** 2016-10-27

**Authors:** Marco Tatullo, Grazia Maria Simone, Franco Tarullo, Gianfranco Irlandese, Danila De Vito, Massimo Marrelli, Luigi Santacroce, Tiziana Cocco, Andrea Ballini, Salvatore Scacco

**Affiliations:** 1 Tecnologica Research Institute, Biomedical Section, Crotone, 88900, Italy; 2 Department of Basic Medical Sciences, Neuroscience and Sense Organs, University of Bari “Aldo Moro”, Bari, 70124, Italy; 3 Unit of Maxillofacial Surgery and Experimental Medicine, Calabrodental, Crotone, 88900, Italy; 4 Marrelli Hospital, Advanced Diagnostic Labs, Crotone, 88900, Italy; 5 Jonian Department DISGEM, University of Bari “Aldo Moro”, Taranto, 74100, Italy

## Abstract

There is increasing interest in identifying natural bioactive compounds that can improve mitochondrial functionality and regulate apoptosis. The brewery industry generates wastewater that could yield a natural extract containing bioactive phenolic compounds. Polyphenols act as antioxidants and have been documented to protect the human body from degenerative diseases such as cardiovascular diseases or cancer. The main aims of our research were to determine the phenolic profile of a crude extract obtained (at pilot scale) from a brewery waste stream and to evaluate the biochemical activity of this extract on the mitochondrial function of a cancer cell line (SH-SY5Y). This work is a basic translational pilot study. The total phenolic content was determined by the Folin–Ciocalteu assay, which revealed that 2.30% of the extract consisted of phenolic compounds. The polyphenols, identified and quantified by reverse-phase-high-performance liquid chromatography and mass spectrometry (RP-HPLC/MS), were mainly flavonoids. After cell culture, the tumoral cells treated with the polyphenolic extract showed enhanced mitochondrial oxidative function, which is likely related to a decrease in oxidative stress and an increase in mitochondrial biogenesis. This type of brewery waste stream, properly treated, may be a promising source of natural antioxidants to replace the synthetic antioxidants currently used in the food industry.

Phenolic compounds differ structurally from simple molecules, such as phenolic acids, and from highly polymerized compounds, such as proanthocyanidins (tannins), which occur in plants and are common in many foods (fruits, vegetables, cereal grains) and beverages (wine, beer, teas)^1^. These compounds present an aromatic ring bearing one or more hydroxyl groups, and their structures may range from a simple phenolic molecule to a complex high-molecular mass polymer^2^. Depending on the number of these phenol rings and on the structural elements bound to them, polyphenols are classified into different groups, namely the flavonoids, phenolic acids, phenolic alcohols, stilbenes and lignans^3^.

Polyphenols provide health benefits through several mechanisms, including the elimination of free radicals and the protection and regeneration of other dietary antioxidants (e.g., vitamin E). Therefore, the study of the functional properties of polyphenolic compounds is an important subject that is relevant to consumers’ food choices. Although bitterness in foods is usually unpleasant, there are some food products such as red wine and beer in which this sensory characteristic is desired^4^,^5^.

Beer contains a large variety of phenolic components derived from the biotechnological fermentation of barley malt (70%) and hops (30%) that are responsible for the overall antioxidant activity of the beverage^6^,^7^. In addition, the polyphenols in beer are important for its chemical stability and include prenylated flavonoids, phenolic acids, simple phenols, hydroxycoumarins, flavones, proanthocyanidins, tannins, and amino phenolic compounds^8^,^9^. These compounds play an important role in flavor (bitterness, astringency, harshness) and colour. The majority of polyphenolic compounds are derived from the malt, and the remainder come from the hops^10^. These compounds are involved in the chemical stability and shelf life of beer. Phenolic compounds can also function as antioxidants in the human body, acting as protective agents against the oxidation of ascorbic acid and unsaturated fatty acids^9^. There have been several studies on the antioxidant activity and phenolic content of beer^11^,^12^. However, this research focused on the relationship between antioxidant activity and total phenolic content, whereas limited data are available on the phenolic profiles and their contribution to antioxidant activity in commercial beers^13^. Moreover, it is difficult to compare data within the literature due to the lack of agreement on the appropriate method for analyzing phenolic compounds and evaluating antioxidant activity. As a consequence, the information in the literature on the levels and species of phenolic compounds is insufficient and contradictory.

Waste materials such as brewery waste streams^14^,^15^ or the vegetative waste material of hops pellets^16^ are rich and currently underused sources of phenolic antioxidants, which could be reused for many industrial or pharmaceutical purposes.

Hence, although there have been numerous studies previously^17^,^18^,^19^, the first objective of this study was to investigate the presence of bioactive compounds in the wastewater obtained during beer production, which can vary from source to source commercially and to identify the range of phenolic compounds.

Human neuroblastoma (SH-SY5Y) is a cell line commonly used in studies related to neurotoxicity, oxidative stress, neurodegenerative diseases, and drug screening^20^,^21^,^22^,^23^. An important second aim is to extend the study to determine the *in vitro* effectiveness of these isolated phenolic compounds on mitochondrial function in the SH-SY5Y cancer cell line^22^,^23^,^24^,^25^.

## Results

**Figure 1:**
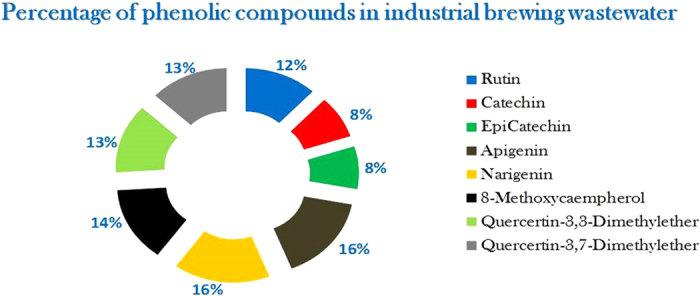
Identification of phenolic compounds in industrial brewery wastewater obtained by the combined method RP-HPLC-DAD. The wastewater is rich in several phenolic compounds, the largest proportions being apigenin and narigenin, two flavonoids particularly active in cancer prevention.

It is well known that phenolic compounds contribute directly to the antioxidant activity of natural extracts, and therefore it is important to evaluate the content of phenolic compounds in the crude extract obtained from wastewater processing^14^,^15^,^16^. In this light, it is important to separate, identify and quantify individual phenolic compounds to characterize the crude extract and to reveal the phenolic profile (Fig. 1). The results obtained from the assessment of the total content of phenolic compounds, expressed as gallic acid equivalents, are reported in Table 1.

**Figure 2:**
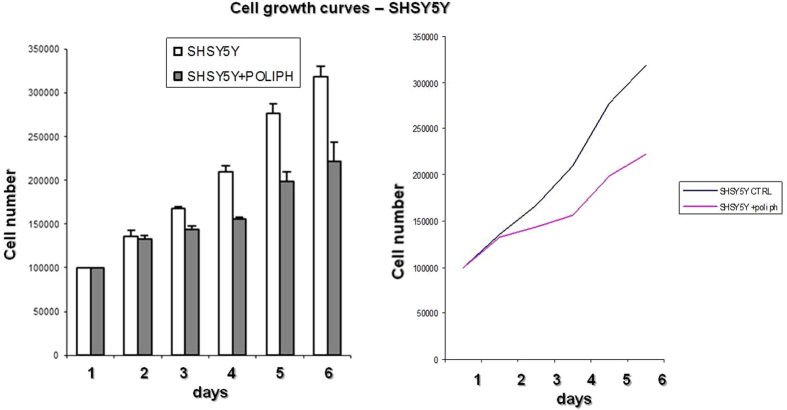
Cell growth trend of the SH-SY5Y line treated and not treated with polyphenolic extracts. The figure shows how SH-SY5Y cell growth is inhibited by the polyphenolic extracts in the culture medium, highlighting the role of the polyphenols in inhibiting the growth of cells of the SH-SY5Y cell line. Experiments were performed in triplicate.

In recent years, apoptosis has become a challenging area of biomedical research. Many molecules that work as cancer-prevention agents induce apoptosis; conversely, several tumor promoters inhibit apoptosis. Therefore, it is reasonable to assume that chemopreventive agents such as phenolic compounds, which have proven effects in animal tumor bioassay systems or human epidemiologic studies on the one hand and induce the apoptosis of cancer cells on the other hand, may have wider implications for the management of cancer^21^. To initiate pilot studies involving phenolics from beer SH-SY5Y cells were accordingly incubated with fresh media containing 1:5 polyphenolic fraction or with an equivalent volume of dimethyl sulfoxide (0.02% DMSO, vehicle) for 6 days to establish whether these compounds actually affect the metabolism/activities of such cancer cells. A significant reduction of cell growth was observed for the cells treated with the polyphenolic compounds (Fig. 2).

**Figure 3:**
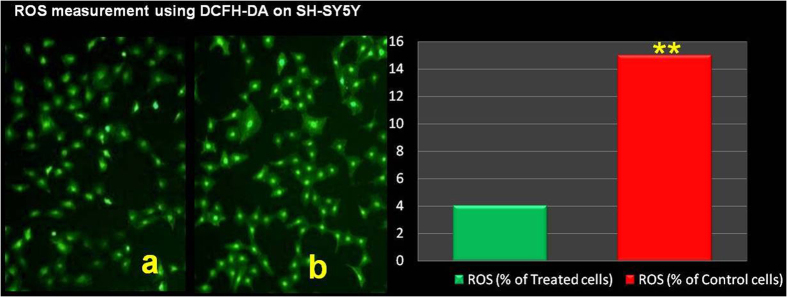
Intracellular ROS content. Treated (a) and control (b) SH-SY5Y cells were exposed to DCFH-DA for 30 min and analyzed by confocal microscopy, as described in the methods section. Treated SH-SY5Y cells were previously incubated for 48 h with polyphenols. Confocal microscopy images of treated SH-SY5Y cells, cultured with polyphenolic compounds extracted from wastewater from the brewery process (a), showed a significantly lower level of DCF-fluorescence, a commonly used probe to assess the intracellular redox state, compared with the control SH-SY5Y cells (b), highlighting the ability of the polyphenols to inhibit intracellular ROS content.

As shown in Fig. 3, the confocal microscopy images of treated SH-SY5Y cells (cultured with polyphenolic compounds extracted from brewery wastewater) revealed a significantly lower level of DCF-fluorescence, a commonly used probe to assess the intracellular redox state, compared with the control cells.

**Figure 4:**
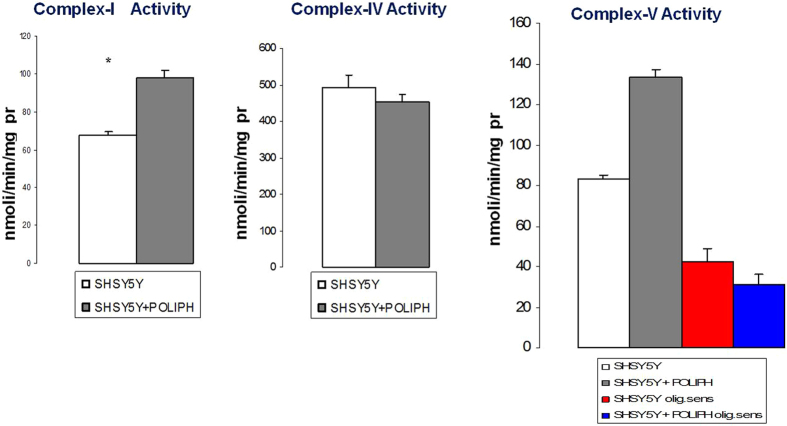
Enzymatic activity of mitochondrial Complex I, IV and V in SH-SY5Y cells untreated and treated with polyphenolic extracts. The enzymatic activity levels of Complex I and V showed interesting variations in SH-SY5Y cells treated with polyphenolic compounds. The activity of CI in polyphenol-treated SH-SY5Y cells increased compared to untreated cells; very similar behavior was shown by the CV ATP-hydrolase activity in the polyphenol-treated cells. CIV did not show any significant variation in activity between treated and untreated cells. Experiments were performed on five replicates each.

Mitochondrial ROS (reactive oxygen species) production in cells is frequently associated with defective activity of respiratory chain complexes, with Complex I (CI) recognized as the major “ROS-genic” site. To verify the insights regarding the effect of the phenolic compounds on the cellular respiratory system, the specific enzymatic activity of mitochondrial Complex I (CI) and Complex IV (CIV) and the ATP hydrolase activity of Complex V (CV) were measured (Fig. 4). The SH-SY5Y cells treated with polyphenolic extracts exhibited variations in the levels of enzymatic activity of Complex I and V compared to untreated tumor cells. The activity of CI in the treated cells was increased compared to cells not treated with our polyphenolic extracts, and a similar trend was detectable for the CV ATP-hydrolase activity in the treated cells. On the other hand, we did not record a significant difference in the activity of CIV between treated and untreated cells. ROS production contributes to mitochondrial damage in several pathologies, and consequently, the behavior of SH-SY5Y cultured with polyphenolic compounds extracted from brewery wastewater that we have reported herein may represent an important therapeutic resource for many of the most investigated diseases, especially cancers.

**Figure 5:**
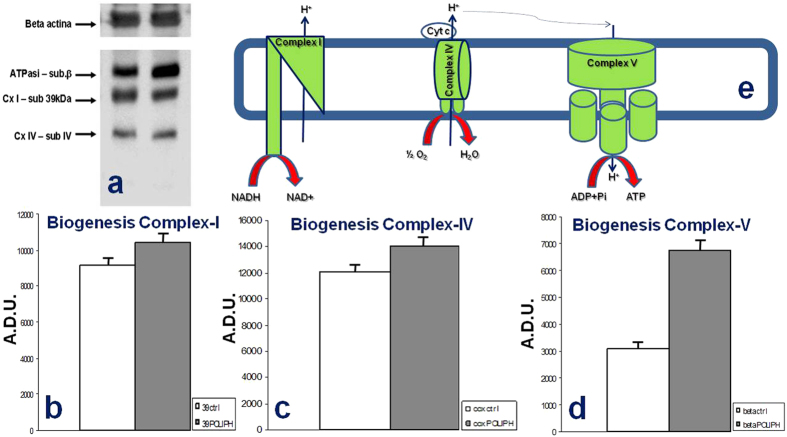
Activity of mitochondrial Complex I (CI), Complex IV (CIV) and Complex V (CV). (**a**) Representative Western blot performed on SH-SY5Y whole cell lysates from control cells and treated cells exposed to beer polyphenols for 8 h, along with the β-actin level, used as a loading control. (**b–d**) Densitometric analysis against three different mitochondrial Complexes (subunit b for ATPase; 39 kDa subunit for Complex I; subunit IV for Complex IV), CTRL *vs.* Treated (p < 0.005). Experiments were performed on five replicates each. (**e**) Schematic representation of Complex I-IV-V.

Finally, to evaluate whether the phenolic fraction increased mitochondrial biogenesis, we measured the mitochondrial DNA (mtDNA) content. As shown in Fig. 5, treatment induced a small but significant increase in the relative mtDNA content (mtDNA/nDNA) measured by q-PCR of the mtDNA cytochrome *b* gene in the treated cells compared to the controls. This confirms that overall the changes observed in mitochondrial activities are not attributable significantly to changed mitochondrial content. These results showed the potential use of such waste products to further explore their use as a source of antioxidants, even though our study reports only preliminary data.

## Discussion

Food waste is a reservoir of complex carbohydrates, proteins, lipids, and nutraceuticals and can provide raw materials for commercially important metabolites^26^,^27^,^28^.

The current legislation on food waste treatment gives high priority to the prevention of waste generation with the least emphasis on disposal. The final and least desirable options are incineration and landfilling^29^,^30^,^31^,^32^. Recent valorization studies on waste from the food supply chain open avenues for the production of biofuels, enzymes, bioactive compounds, biodegradable plastics, and nanoparticles, among many other molecules^28^.

Polyphenols are bioactive compounds and have been reported to potentially exert anticancer effects via a variety of mechanisms, including the removal of carcinogenic agents^28^,^29^, modulation of cancer cell signaling^33^,^34^ and cell cycle progression^35^,^36^, promotion of apoptosis^37^,^38^,^39^ and modulation of enzymatic activities^40^.

In this study, a new by-product (wastewater derived from beer production) was found to be a natural source of bioactive compounds with several potential applications.

In industrial brewing, approximately one liter of wastewater is generated for every 138 L of beer produced. Therefore, considering the amounts of beer produced by the top beer producing regions in 2010, Europe (403 million hectoliters), China (466 million hectoliters) and the United States (207 million hectoliters), up to 434, 502 and 223 tons of crude extract could be obtained in these regions, respectively^29^,^30^.

The selection of an appropriate extraction method depends mainly on the advantages and disadvantages of the processes, such as extraction yield, complexity, production cost, environmental friendliness and safety.

Similar studies have been performed on similar topics. An *in vitro* study on the antioxidant and antimicrobial potential of the phenolic extracts from olive mill wastewater was recently reported in the literature; antioxidant activity was demonstrated for all the tested phenolic olive mill wastewater extracts, and moreover, they all showed significant antibacterial activity against *Staphylococcus Aureus*^41^.

Some studies have investigated the potential reuse of waste from fruit, assuming that fruit waste might have antioxidant activity. A recent study focused on the antioxidant activity of waste obtained in the production of pineapple products; a peroxide value analysis reported that pineapple waste extracts induced a reduction in oxidation products, confirming the antioxidant power of the extracts obtained from this fruit^42^.

While several studies have analyzed the antioxidant activity of the polyphenolic extracts derived from different sources, few studies have focused on the antitumor activity of these compounds. An interesting study investigated the antitumor effects against HeLa cells of a polyphenol-rich extract from *Pinus sylvestris* L. bark: in this study, pine bark polyphenolic extracts exhibited high cytotoxicity against HeLa cells, highlighting their ability to induce HeLa cell apoptosis^43^.

Our study describes for the first time the antioxidant and antitumor activity of the bioactive polyphenolic fraction isolated from the beer brewing process; this work is a pilot study to highlight significant and useful data that could lead to the worldwide collection of wastewater from the brewing process. We believe that this study is the first step to standardizing the extraction of polyphenolic compounds, leading to further industrial and pharmaceutical applications.

In conclusion, new possibilities for these natural extracts can be further investigated, specifically in relation to their antioxidant and antitumor activities.

## Methods

### Determination of total phenolic content

The total content of phenolic compounds in the crude extract was determined according to the Folin–Ciocalteu colorimetric method described by Singleton, Orthofer, and Lamuela-Raventos (1999), with some modifications^22^,^23^.

An aliquot (100 μL) of sample solution was mixed with 3.75 mL of ultrapure water in a test tube, and 0.5 mL of Folin–Ciocalteu reagent (diluted 1:1 (v/v) with ultrapure water) was added. After 2 min, 0.5 mL of 20% sodium carbonate anhydrous solution was added to the mixture.

The solution was allowed to stand for 1 h at room temperature, and the absorbance of the resulting blue complex was measured at 765 nm in a dual-beam spectrophotometer (Uvikon XL, Bio-Tek Instruments, Milan, Italy).

All determinations (10 samples) were performed in triplicate. Quantification was based on the standard curve of commercial gallic acid, and the concentration of total phenolic compounds was expressed as mg of gallic acid equivalents (GAE)/g of dry extract.

The brewery wastewater was kindly supplied by a brewing company in Italy.

### Separation and Quantification of Bioactive Phenolic Compounds (RP-HPLC-DAD)

Chromatographic analysis was performed on an high-performance liquid chromatography (HPLC) system model 1200HP (Hewlett-Packard, Waldbronn, Germany), equipped with a Diode Array Detector (DAD) and controlled by the HP Chemstation chromatography software. The chromatographic separation of polyphenols was performed on a reverse phase Kromasil C18 column (250 × 3.2 mm internal diameter, 5 μm particle size) (Phenomenex, Barcelona, Spain). The solvents constituting the mobile phase were 0.1% acetic acid in Milli-Q water (solvent A) and 100% ACN (solvent B). The gradient program was as follows: 0–5 min, 90% A and 10% B; 5–35 min, linear gradient until reaching 50% B at 35 min; 35–43 min, 50% B isocratic; 43–45 linear gradient from 50% to 10% B; and finally, the column was washed and reconditioned. The mobile phase flow rate was 0.5 mL/min^−1^ during the entire analytical run, the column temperature was set at 38 °C, and the sample injection volume was 20 μL. A scan in the range of 190 to 700 nm was continuously performed by DAD. Individual phenolic compounds were identified by comparing their retention times and UV spectra with those obtained by injecting standards under the same HPLC conditions.

### Culture conditions

SH-SY5Y cells were purchased directly from ATCC (CRL-2266). SH-SY5Y cultures were grown as described previously by Jordán *et al.*^24^ in Dulbecco’s modified Eagle’s medium (DMEM) supplemented with 2 mM L-glutamine, 20 units·mL^−1^ penicillin, 5 mg·mL^−1^ streptomycin, and 15% (v/v) fetal bovine serum (Invitrogen, Carlsbad, CA, USA). The SH-SY5Y cells (1 × 10^6^/mL) were seeded 24 h before the experiments in a 96-well plate and grown in a humidified cell incubator at 37 °C under a 5% CO2 atmosphere.

For treatments, extracts from beer wastewater were directly added to the culture medium at different concentrations (0.1, 0.25, 0.50, 0.75, and 1 mg/mL). The corresponding controls were treated with the same concentration of ethanol, which was always below 0.1% (final concentration).

For treatment conditions, the medium was removed, and the cells were incubated with fresh media containing 1:5 polyphenolic fraction or with an equivalent volume of dimethyl sulfoxide (0.02% DMSO, vehicle) for 6 days (Fig. 2). In the time/response treatments, a parallel experiment exposing the cells to DMSO was used as a control to calibrate the observed results.

The methods were performed in accordance with the relevant guidelines and regulations.

### Real-time PCR

The total RNA was purified from SH-SY5Y cells using the RNeasy Mini Kit (Qiagen S.r.l., Milano, Italy), according to the manufacturer’s protocol. One microgram of total RNA was then reverse-transcribed to generate the cDNA for PCR using the iScript cDNA Synthesis kit (Bio-Rad Laboratories). The q-PCR on cDNA was performed as previously described^25^, using glyceraldehyde-3-phosphate dehydrogenase (GAPDH) and β-actin as internal controls.

The mtDNA content was assayed by q-PCR using 100 ng of total DNA, isolated using a DNA extraction kit (EuroGold Tissue DNA Mini Kit), with primers amplifying the cytochrome b-region and normalized to the 18S nuclear DNA. Relative quantification was performed using the ΔΔ*C*T method.

### Quantitative determination of intracellular ROS level

The ROS level was quantitatively determined using the cell permeant probe 2′-7′dichlorodihydrofluorescin diacetate (H2DCFDA). The ROS-dependent oxidation of the fluorescent probe (507 nm excitation and 530 nm emission wavelengths) was measured by a Jasco FP6200 spectrofluorometer as previously described^25^.

### Laser scanning confocal microscopy

Laser scanning confocal microscopy (LSCM) live cell imaging of ROS was performed in cells cultured at low density on fibronectin-coated 35-mm glass-bottom dishes incubated for 20–30 min at 37 °C with 10 μM 2,7-dichlorofluorescin diacetate, which is converted to dichlorofluorescein by intracellular esterases, or with 5 μM MitoSOX (Molecular Probes, Eugene, OR).

Stained cells were washed with PBS and examined with a Nikon TE 2000 microscope (images collected using a 60 × objective [1.4 NA]) coupled to a Radiance 2100 dual-laser LSCM system (Bio-Rad Laboratories, Milan, Italy); dichlorofluorescein green fluorescence was elicited using the Ar–Kr laser beam (λex 488 nm), and MitoSOX red fluorescence was elicited using the He–Ne laser beam (λex 543 nm).

Data acquisition, storage, and analysis were performed using the LaserSharp and LaserPix software from Bio-Rad or ImageJ version 1.37.

For fluorescence microscopy analysis, SH-SY5Y cells were washed twice in PBS and incubated with the CYTO-ID Green Detection Reagent and Hoechst 33342 Nuclear Stain (CYTO-ID Autophagy Detection Kit, Enzo Life Science, NY, USA) according to the manufacturer’s instructions, mounted in VECTASHIELD (Vector Laboratories, Inc., Burlingame, CA) and examined with an Olympus photomicroscope (Olympus Italia, Rozzano, Italy) using 63× objective lenses with either 1× zoom factor.

Images were analyzed, digitally recorded and stored as TIFF files using the Adobe Photoshop software (Adobe Systems Inc., San Jose, CA, USA).

Morphometric analysis of the labeled areas was performed on twenty randomly selected fields for each experimental group, observed at × 630 magnification with an Olympus photomicroscope, using the Image Analysis software (Olympus Italia, Rozzano, Italy).

#### Statistical analysis

Analysis of variance and multivariate analysis were performed using the SPSS 15.0 for Windows statistical package. The significance of the differences among means was determined using Student’s t test. A *p* value < 0.05 was defined as a statistically significant difference.

## Additional Information

**How to cite this article**: Tatullo, M. *et al.* Antioxidant and Antitumor Activity of a Bioactive Polyphenolic Fraction Isolated from the Brewing Process. *Sci. Rep.* **6**, 36042; doi: 10.1038/srep36042 (2016).

**Publisher’s note:** Springer Nature remains neutral with regard to jurisdictional claims in published maps and institutional affiliations.

## Figures and Tables

**Table 1:** The concentration of total phenolic compounds was expressed as mg of gallic acid equivalents (G.A.E.)/g of dry extract.

Sample Number	mg of G.A.E.
1	13.81
2	13.78
3	13.80
4	13.81
5	13.79
6	13.76
7	13.77
8	13.75
9	13.82
10	13.83
